# Monoclonal and Bispecific Anti-BCMA Antibodies in Multiple Myeloma

**DOI:** 10.3390/jcm9093022

**Published:** 2020-09-19

**Authors:** Benedetta Dalla Palma, Valentina Marchica, Maria Teresa Catarozzo, Nicola Giuliani, Fabrizio Accardi

**Affiliations:** 1 Department of Medicine and Surgery, University of Parma, 43126 Parma, Italy; valentina.marchica@unipr.it (V.M.); mariateresa.catarozzo@gmail.com (M.T.C.); nicola.giuliani@unipr.it (N.G.); 2 Hematology and BMT Unit, Azienda Ospedaliero-Universitaria di Parma, 43126 Parma, Italy; 3 Department of Hematology I, Azienda Ospedaliera Ospedali Riuniti Villa Sofia-Cervello, 90146 Palermo, Italy

**Keywords:** multiple myeloma, B-cell maturation antigen, monoclonal antibody treatment

## Abstract

B-cell maturation antigen (BCMA), a member of the tumor necrosis factor receptor superfamily, is universally expressed by normal and neoplastic plasma cells and plays a critical role in the proliferation, survival and tumor progression in multiple myeloma (MM). B-cell activating factor (BAFF) and a proliferation-inducing ligand (APRIL) have been recognized as proliferation ligands for BCMA in the bone marrow microenvironment. Soluble BCMA levels in the serum correlates with disease phase and tumor burden and is a predictor of progression-free survival (PFS) and overall survival (OS). Recently, the introduction of new monoclonal antibodies against CD38 (Daratumumab and Isatuximab) and SLAM7 (Elotuzumab) has changed the therapeutic approach to MM, improving the response rate and the time to progression, both in newly diagnosed and refractory/relapsed patients. Among the surface antigens on MM cells, BCMA is a suitable target for the design of new antibody-based strategies. Experimental approaches targeting BCMA are currently being investigated and include antibody-drug conjugates (ADCs), bispecific antibodies (bsAbs) and genetically engineered T-cells with chimeric antigen receptors (CAR). In this review we summarize the more recent findings about BCMA biologic rationale as a therapeutic target and report the updated results of preclinical and clinical studies focused on ADCs and bsAbs targeting BCMA.

## 1. Introduction

Multiple myeloma (MM) cells are characterized by a tight relationship with the bone marrow (BM) microenvironment cells, which are critically involved in MM cell survival, apoptosis and drug resistance. Several surface molecules expressed by MM cells that are primarily involved in cell adhesion are suitable targets for the use of specific antibodies against these antigens [1,2,3,4].

The use of therapeutic monoclonal antibodies (mAbs) has been recently introduced in the treatment of relapsed/refractory MM (RRMM) patients [5,6].

The mAbs anti-CD38 (Daratumumab) and anti-SLAMF7 (Elotuzumab) were the first to be approved in RRMM patients [7]. Moreover, the efficacy of these mAbs was significantly improved by combining them with other anti-MM drugs such as the immune-modulatory drugs (IMiDs) lenalidomide and pomalidomide [8,9,10].

The anti-MM effect of the anti-CD38 mAb Daratumumab was also potentiated by the combination with proteasome inhibitors (PIs), such as Bortezomib and Carfilzomib [11,12]. The use of these mAbs has changed the therapeutic approach in MM patients, improving the response rate and the time to progression. Both Daratumumab and Elotuzumab are being tested in clinical trials as a first line of treatment, in combination with PIs and IMiDs [13,14].

With the introduction of the mAbs in MM therapy, an increasing number of preclinical studies have been investigating possible new surface antigens on MM cells, which could be targeted by new mAbs. Moreover, preclinical research has also moved to an alternative approach based on the design of engineered bispecific antibodies.

Of the surface molecules overexpressed by MM cells, the B-cell Maturation Antigen (BCMA) is the most promising antigen, and therapeutic antibodies against BCMA have been rapidly developed with different technologies [15,16].

In this review we describe the biological role of BCMA in MM cells, and summarize the main anti-BCMA therapeutic antibodies and their clinical results in MM patients.

## 2. Biologic Role of BCMA in MM Cells

BCMA, also known as TNFRSF-17, transmembrane activator and calcium modulator and cyclophilin ligand interactor (TACI) and B-cell activating factor receptor (BAFF-R, synonyms BR3 and TNFRSF-13C) are all members of the tumor necrosis factor receptor (TNFR) superfamily. These receptor subtypes are expressed differentially by primary MM cells and human myeloma cell lines (HMCLs). BCMA is expressed by all MM cells, TACI expression is heterogeneous among primary samples and HMCLs, while BAFF-R is lacking in HMCLs and partially expressed in primary myeloma cells [17,18,19,20].

Two different soluble growth factors in the BM microenvironment, BAFF and A proliferation-inducing ligand (APRIL), have been identified as proliferation ligands for these receptors, and play a critical role in the proliferation differentiation and survival of B-cells and plasma cells [21].

BAFF can form homotrimers, heterotrimers with APRIL or multimers. The level of BAFF is higher in MM patients compared to healthy donors and correlates with prognostic markers, disease burden and angiogenesis in BM [22,23,24]. Furthermore, BAFF affinity is 1000-fold higher for BAFF-R compared to BCMA, while APRIL shows a higher selectivity for BCMA and TACI. In the latter case, the binding is mediated by CD138/syndecan-1 [20]. BAFF and APRIL ligation to these receptors induces the activation of NF-κB pathways and the upregulation of antiapoptotic proteins (Mcl-1, Bcl-2, Bcl-xL) revealing how the BAFF/APRIL axis maintains a crucial role supporting neoplastic plasma cells (PCs) survival [19,22].

Moreover, these ligands are involved in drug resistance, therefore rescuing HMCLs from dexamethasone-induced apoptosis and IL-6 deprivation [22].

Osteoclasts, monocytes and myeloid cells are the main contributors to APRIL and BAFF production in MM BM microenvironments via a paracrine effect, but a minor role of MM cells via autocrine stimulation has also been reported [25]. 

BCMA as a receptor showed a critical role in the survival of long-lived PCs, however it is not required in B-cell differentiation and homeostasis, as demonstrated in a BCMA knock-down mouse model [26]. BCMA is more highly expressed in neoplastic plasma cells compared to healthy donors and at a minor level on plasmacytoid dendritic cells (pDC) [27]. Conversely, it is absent on naïve and memory B lymphocytes, T-cells and other nonlymphoid organs [28,29].

Soluble BCMA (sBCMA) is derived from the ectodomain shedding of the membrane receptor by γ-secretase (GS) [30]. The intramembranous protease GS cleaves the extracellular domain and releases sBCMA in biological fluids. In contrast to membrane-bound BCMA, which binds both BAFF and APRIL, soluble BCMA (sBCMA) is a decoy receptor and seizes mostly APRIL. In vivo, the inhibition mediated by GS increases BCMA surface expression and the number of PCs in BM [30]. sBCMA is correlated with BM PCs infiltration and disease phases, as increased levels are reported through the progression from monoclonal gammopathy of undetermined significance (MGUS) to smoldering MM and active MM [31]. Moreover, sBCMA levels are correlated with clinical responses according to International Myeloma Working Group (IMWG) criteria. MM patients with sBCMA levels above the median (326.4 ng/mL) had significantly shorter progression-free (3.6 months) and overall survival (OS) (98 months) than patients whose levels were below the median (9.0 and 155 months, respectively) [32].

## 3. BCMA as a Therapeutic Target

BCMA overexpression by HMCLs in a xenograft model increases cell proliferation and upregulates the expression of genes correlated with angiogenesis, adhesion, osteoclast activation and immunosuppression [19].

Furthermore, sBCMA, acting as a decoy receptor, has been identified as an inhibitor of B-cell differentiation and involved in immunoparesis which is a hallmark of MM and correlated with a worst prognosis and tumor progression [33,34].

The administration of recombinant human BCMA (rhBCMA) reduces plasma BAFF levels, and as a consequence, decreases immunoglobulins levels in immunocompetent mice. In MM patients, BCMA-BAFF complex in a serum has been inversely correlated to uninvolved immunoglobulin level through a mechanism based on BAFF trapping and the consequent reduced stimulation and differentiation of polyclonal B-cells [33].

BCMA antibodies, able to induce antibody-dependent cell-mediated cytotoxicity (ADCC) mediated lysis in BCMA expressing cells, were identified in the serum of allotransplanted MM patients after donor lymphocyte infusion, thus demonstrating the role of BCMA as target for graft versus tumor response [35].

MM is a very heterogeneous disease and the loss or downregulation of surface antigens, including BCMA, secondary to clonal evolution, has been reported [36,37]. In an MM mouse model, a small oral GS inhibitor (GSI) LY3039478 induced an increase of BCMA expression on MM cells by inhibiting the release of sBCMA in peripheral blood, improving the efficacy of adoptive T-cell therapy targeting BCMA. Moreover, these results have been confirmed in a small group of MM patients, where short-term exposition to GSI increases the percentage of BCMA positive MM cells [38].

**Figure 1:**
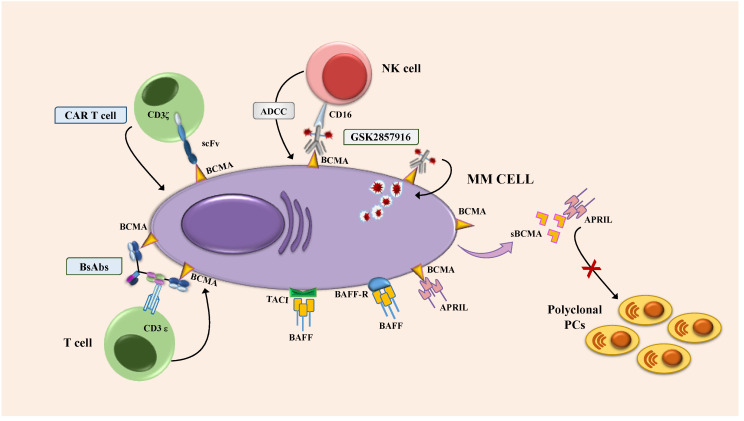
Immunotherapeutic approaches targeting B-cell maturation antigen (BCMA). Abbreviations: ADCC: antibody-dependent cellular cytotoxicity; APRIL: A proliferation inducing ligand; BAFF: B-cell activating factor; BAFF-R: BAFF receptor; BCMA: B-cell maturation antigen; BsAbs: bispecific antibodies; CAR T: chimeric antigen receptor T-cell; scFv single-chain variable fragment. MM: multiple myeloma; NK: natural killer; PC: plasma cells; TACI: transmembrane activator and CAML interactor.

In conclusion, these scientific works demonstrated that BCMA is an ideal target for the design of new innovative approaches in MM (Figure 1). Innovative experimental therapies targeting BCMA are being increasingly used in clinical practice and include antibody-drug conjugates (ADCs), bispecific antibodies (bsAbs) and genetically engineered T-cells with chimeric antigen receptor (CAR). The last therapeutic approach is well summarized in other reviews [4,39].

## 4. Investigational Antibody-Based Approach Targeting BCMA

### 4.1. Antibody-Drug Conjugate: Mechanism and Preclinical Model

ADCs are complex molecules, composed by three components: *(i)* a tumor-associated antigen (TAA)-targeted mAb; *(ii)* a cytotoxic drug, e.g., the tubulin polymerization inhibitors monomethyl auristatin F (MMAF) or monomethyl auristatin E (MMAE), the DNA double-strand breaking agent calicheamicin, pyrrolobenzodiazepine (PBD), which are DNA minor-groove crosslinking agents, or other toxins; *(iii)* a linker that joins the two other components [40]. Once the mAb binds to its specific target cell, ADC is internalized, and the cytotoxic drug is released to induce cell damage and death. 

The first anti-BCMA antibody developed as naked antibodies and as antibody-drug conjugates, named cSG1, showed strong cytotoxic activity against HMCLs [41].

GSK2857916 (Belantamab Mafodotin) is the first anti-BCMA ADC that has been investigated in clinical trials. It is an afucosylated, humanized IgG1 mAb conjugated with monomethyl auristatin F (MMAF), a tubulin polymerization inhibitor, through a noncleavable linker with a high affinity for BCMA [27]. This antibody–drug conjugate displays enhanced killing and decreased toxicity through three known mechanisms. The first, the ADCC effect, mediated by NK cells, which is increased by defucosylation of the constant region fragment (Fc) carbohydrates. In the second, after lysosomal endocytosis, the toxin MMAF is released in the cytoplasm where it inhibits microtubule polymerization and induces apoptosis. Last of all, the noncleavable maleimidocaproyl linker prolongs the stability of the antibody–drug conjugate in the blood with limited toxicity on off-target cells [27].

In a preclinical study, GSK2857916 demonstrated all of these properties by selectively killing MM cells (either primary MM cells or HMCLs), alone or in coculture with BM stromal cells. The same study demonstrated, in a xenograft mouse model, that treatment with this ADC can induce tumor regression for up to 3.5 months [27].

Other ADCs have been tested in preclinical MM models. Among those, MEDI2228 is a fully human antibody conjugated to a PBD dimer via a protease-cleavable linker; the drug has shown antimyeloma activity in preclinical models, both in primary myeloma progenitors cells and BCMA-positive HMCLs [42].

HDP-101 is an anti-BCMA antibody conjugated to amantin; amantin is a toxin of the amatoxin family that binds to the RNA polymerase II, inhibiting the cellular transcription process. [43] HDP-101 has demonstrated activity in preclinical models against MM cells harboring del(17p) abnormality, where a reduced RNA polymerase II subunit A expression is observed [44].

#### 4.1.1. Clinical Trials, Patient Population and Efficacy

Belantamab Mafodotin was the first BCMA antibody-drug conjugate tested in clinical trials. The phase I trial (BMA117159) was conducted in heavily pretreated patients: 89% were double refractory to both PIs and IMiDs and 37% were refractory to Daratumumab [45]. The updated and recently published results showed a 60% Overall Response Rate (ORR), with the majority of patients (19 out of 21) achieving a Very Good Partial Response (VGPR) or better; a response was observed also in patients triple-refractory to PIs, IMIDs and Daratumumab (ORR 38.5%). Median progression-free survival (PFS) in the whole population was 12 months, and median duration of response (DOR) was 14.3 months; median PFS in patients treated with Daratumumab and refractory to both IMIDs and PIs was 6.2 months [46].

Belantamab Mafodotin was therefore tested as a single agent in a randomized phase II study (DREAMM-2), which enrolled 196 patients in two different cohorts (which tested 2 doses of the drug, 2.5 mg/kg and 3.4 mg/kg every 3 weeks). The patients selected had a median of previous line of therapy of 6 (range 3–21) and 7 (range 3–21) in the two cohorts, respectively; all patients were refractory to IMIDs and PIs, and had been treated with anti-CD38 monoclonal antibodies. An overall response was reached in 31% of patients in the 2.5 mg/kg cohort, and in 34% of patients in the 3.4 mg/kg cohort [47]. A recent update of the study, with a median follow-up of 9 months, confirmed the ORR data and showed a median PFS of 2.8 and 3.9 months in the two different cohorts, respectively; 1-year overall survival (OS) probability was 53% [48]. Response rates were similar even when stratifying patients by number of prior therapies: 32% in patients with 3–6 previous lines and 30% in patients with ≥7 prior lines of therapy [49].

Other studies with Belantamab Mafodotin either as single-agent or in combination with other drugs are currently ongoing. Worthy of note, the preliminary data of the DREAMM-4 study, a phase I/II study of Belantamab Mafodotin at 2 different dosages (2.5 and 3.4 mg/kg every 3 weeks), in combination with Pembrolizumab in RRMM patients has been recently presented. 13 patients have been enrolled so far. The combination showed clinical activity, with an ORR of 67% in the 2.5 mg/kg arm and of 14% in the 3.4 mg/kg arm [50]. Available results of the clinical trials with Belantamab Mafodotin are summarized in Table 1. Belantamab Mafodotin is also being tested in earlier lines of treatment. Particularly, a phase I trial (NCT04091126) is currently ongoing, testing the association of Belantamab Mafodotin with Bortezomib, Lenalidomide and Dexamethasone (see Table 2), but results have not been reported yet.

Belantamab Mafodotin has been recently approved by the US Food and Drug Administration (FDA) as a monotherapy treatment for adult patients with RRMM who have received at least four prior therapies, including an anti-CD38 monoclonal antibody, a proteasome inhibitor and an immunomodulatory agent. 

There are many other anti-BCMA ADCs in clinical development. A phase I trial of MEDI228 as a single-agent in RRMM patients is currently ongoing (NCT number: NCT03489525), but results are not yet available. Interestingly, preclinical studies showed that MEDI288 upregulates CD38 expression in MM cells, giving the rationale for a potential combination with anti-CD38 mAbs [51]. CC-99712 is another BCMA ADC, but the drug composition is not available and no preclinical studies have been published; a phase I study in RRMM patients is currently ongoing (NCT number: NCT04036461) but results are yet to be reported.

Ongoing clinical trials with anti-BCMA ADC are summarized in Table 2.

#### 4.1.2. Toxicity

Belantamab Mafodotin has shown a manageable toxicity profile. In the phase I BMA117159 trial, all the patients enrolled (*n* = 35) developed at least one adverse event (AE): the most common AE was thrombocytopenia that occurred in 63% of patients; other recurrent AEs were blurred vision (51%) and cough (40%). Corneal events such as blurred vision, dry eye and photophobia were reported in 69% of patients, mostly grade 1 or 2 (54%). Grade 3 and 4 AEs were reported in 83% of patients and were mostly hematological (thrombocytopenia and anemia). Infusion-related reactions (IRRs) occurred in 12% of patients [46].

In the DREAMM-2 study, the most common grade 3–4 adverse events (AEs) were kerathopathy (27% in the 2.5 mg/kg arm and 21% in the 3.4 mg/kg arm), thrombocytopenia (20% and 33%, respectively) and anemia (20% and 25% in the two different cohorts); IRRs occurred in 21% and 16% of patients in the two arms of treatment, mostly grade 1 or 2 [47].

The combination with Pembrolizumab does not seem to add toxicity during treatment; in the DREAMM-4 study, the main AEs were keratopathy, fatigue and anemia [50].

### 4.2. Bispecific Antibodies

Recently, the anti-BCMA antibody strategy has been enriched by the introduction of bsAbs, which allow an effective T-cell-mediated killing through the direct engagement of BCMA expressing neoplastic plasma cells and effector cells.

BI 836,909, also named AMG420, is the first in class, bispecific T-cell engager (BiTE^®^) (Thousand Oaks, CA, US) constructed as two linked single-chain variable fragments (scFvs) specific for BCMA and CD3ε, respectively [52]. 

A phase I dose escalation (0.2–800 μg/die) trial evaluated AMG 420 treatment in 42 RRMM patients, administered as a continuous infusion due to its low molecular weight and short half-life, for 4 weeks every 6 weeks. An objective response rate of 70% was reported in 10 patients at the maximum tolerated dose of 400 μg/die. Median duration of response was 9 months. In the whole population, two patients died for AEs related to the treatment (influenza/aspergillosis and adenovirus-related hepatitis) and the serious AEs most frequently reported were infections, peripheral neurophaty, edema and cytokine release syndrome (CRS). Central nervous toxicity was mild [53]. 

In order to increase the bsAbs half-life and obtain a better feasibility, new generation BitEs, such as AMG701 and DuoBodies, designed as two antigen binding fragments (Fab) and a constant region fragment, are currently being studied in clinical trials [54].

EM801, also named CC-93269, is a bsAb constructed as an asymmetric two-arm IgG1 humanized antibody with an engineered Fc region leading to a prolonged half-life. One arm shows a low affinity for CD3 on T-cells, mediated by a monovalent binding, to avoid significant toxicity related to excessive T-cell activation. The other arm shows a high affinity for BCMA through a divalent binding. A strong CD4/CD8 T-cell engagement and activation against autologous MM cells with cytotoxic granules secretion has been reported in vitro [55]. A phase 1 study investigating CC-93269 (0.15–10 mg) as a weekly infusion was conducted in heavily pretreated RRMM patients (66.7% triple-class refractory) and showed promising results in terms of efficacy. ORR in all patients was 43.3%, with a sCR/CR of 16.7%; ORR increased to 88.9% (with a sCR/CR rate of 44.4%) in patients receiving the 10 mg dose. CRS was reported in 89.5% and more frequent serious AEs were hematological toxicity and infections [55].

AMG 701 is a new anti-BCMA BiTE characterized by a long half-life (112 h), and in a preclinical model induced a robust activation of CD4/CD8 T enhanced by the combination with IMiDs (lenalidomide or pomalidomide). A clinical trial investigating this agent in RRMM (NCT03287908) is ongoing [56]. 

Usmani et al. recently presented the preliminary results of a phase 1 trial of Teclistamab, a humanized BCMA × CD3 bispecific antibody, tested at the dose of 0.3–270 μg/kg in RRMM patients; 78% of patients showed a response to a weekly treatment at the highest tested dose. Severe neurotoxicity was reported in 3% of patients. Other severe AEs were infections and hematological toxicity [57].

Finally, additional bsAbs currently being tested in clinical trials or at a preclinical level include PF-06863135, REGN-5458, REGN-5459 and TNB-383B [54].

## 5. Conclusions

Preclinical evidence indicates that BCMA is a suitable and new therapeutic target in MM because it is overexpressed by MM cells. Anti-BCMA mABs are currently under clinical evaluation. Clinical data indicate that highly pretreated and refractory MM patients have responded to Belantamab Mafodotin, although the duration of the response is a few months. Belantamab Mafodotin could be a treatment option for MM patients that are refractory to several drugs and lines of treatment, including anti-CD38 monoclonal antibodies. However, it is likely that the best efficacy for this mAb might be observed in combination with other drugs, such as the inhibitors of the PD-1/PD-L1 axis. On the other hand, preliminary clinical data indicate that bispecific anti-BCMA antibodies seem to be promising in RRMM. Future clinical studies of phase II should be conducted to demonstrate the efficacy of this type of approach in MM patients.

## Figures and Tables

**Table 1:** Preliminary results of main clinical trials with GSK2857916 (Belantamab Mafodotin).

TRIAL NAME	NCT Number	PHASE	Number of Patients (Treatment Arm)	Previous Lines of Treatment (Median)	Previous Drugs	Combinatio *n* with Other Drugs	Efficacy	Aes (Grade 3–4)	Reference
BMA117159	NCT02064387	I	35 (part 2)	1–≥10	IMIDs PIs Alkilators	/	ORR 60% Median PFS 12 Months	Corneal Events (9%), Thrombocytopenia (26%), Anaemia (14%)	Trudel S, Blood Cancer Journal (2019) 9:37
DREAMM-2	NCT03525678	II	196	3–21 (7)	IMIDs PIs Anti-CD38	/	ORR: 31% (2.5 mg/kg), 34% (3.4 mg/kg)Median PFS: 2.8 (2.5 mg/kg), 3.9 Months (3.4 mg/kg)	Kerathopathy (27% and 21%) Thrombocytopenia (20% and 33%) Anemia (20% And 25%)	Lonial S, Lancet Oncol 2020; 21:207–221
DREAMM-4	NCT03848845	I/II	13 (part 1)	3–13 (7.5)–2.5 mg/kg cohort 3–8 (5)–3.4 mg/kg cohort	IMIDs PIs Anti-CD38	Pembrolizumab	ORR: 67% (2.5 mg/kg Cohort), 14% (3.4 mg/kg Cohort)	Kerathopathy (33% and 57%)	Nooka AJ, EHA2020, Abs EP955

Abbreviations: IMIDs, immunomodulatory drugs; PIs, proteasome inhibitors; ORR, overall response rate; PFS, progression-free survival

**Table 2:** Ongoing clinical trials with BCMA antibody-drug conjugate.

DRUG NAME	TRIAL NAME	NCT Number	PHASE	Setting	Combination with Other Drugs	Comparator Arm
**GSK2857916**	BMA117159	NCT02064387	I	RRMM	/	/
	DREAMM-2	NCT03525678	II	RRMM	/	/
	DREAMM-3	NCT04162210	III	RRMM	/	Pomalidomide/Dexamethasone
	DREAMM-4	NCT03848845	I/II	RRMM	Pembrolizumab	/
	DREAMM-5	NCT04126200	I/II	RRMM	GSK3174998 GSK3359609 Nirogacestat	/
	DREAMM-6	NCT03544281	I/II	RRMM	Lenalidomide/Dexamethasone Bortezomib/Dexamethasone	/
	DREAMM-7	NCT04246047	III	RRMM	Bortezomib/Dexamethasone	Daratumumab/Bortezomib/Dexamethasone
	DREAMM-8	NCT04484623	III	RRMM	Pomalidomide/Dexamethasone	Pomalidomide/Bortezomib/dexamethasone
	MCRN 007	NCT03715478	I/II	RRMM	Pomalidomide/Dexamethasone	/
	209664	NCT04091126	I	NDMM	Bortezomib/Lenalidomide/Dexamethasone	/
**MEDI2228**	D7900C00001	NCT03489525	I	RRMM	/	/
**CC99712**	CC-99712-MM-001	NCT04036461	I	RRMM	/	/

Abbreviations: RRMM, relapsed/refractory multiple myeloma; NDMM, newly diagnosed multiple myeloma
